# Multimodal assessment of acute cardiac toxicity induced by thoracic radiotherapy in cancer patients. Study protocol

**DOI:** 10.1186/s12885-021-08823-3

**Published:** 2021-10-18

**Authors:** Tomás Merino, Mauricio P. Pinto, María Paz Orellana, Gonzalo Martinez, Marcelo Andía, Pablo Munoz-Schuffenegger, Francisco Acevedo, Luigi Gabrielli, Cesar Sanchez, Jaime Pereira

**Affiliations:** 1 Department of Hematology-Oncology, School of Medicine, Pontificia Universidad Católica de Chile, Santiago, Chile; 2 Division of Cardiovascular Diseases, School of Medicine, Pontificia Universidad Católica de Chile, Santiago, Chile; 3 Department of Radiology, Faculty of Medicine, Pontificia Universidad Católica de Chile, Santiago, Chile; 4 Advanced Center for Chronic Diseases Department of Cardiology, Cardiovascular Division, School of Medicine, Pontificia Universidad Católica de Chile, Santiago, Chile

**Keywords:** Radiotherapy, Cancer, Cardio-oncology, Echocardiography, Magnetic cardiac resonance, Circulating endothelial cells, Predictors, Cardiotoxicity

## Abstract

**Background:**

Today, cancer ranks as one of the leading causes of death. Despite the large number of novel available therapies, radiotherapy (RT) remains as the most effective non-surgical method to cure cancer patients. In fact, approximately 50% of all cancer patients receive some type of RT and among these 60% receive RT-treatment with a curative intent. However, as occurs with any other oncological therapy, RT treated patients may experience toxicity side effects that range from moderate to severe. Among these, cardiotoxicity represents a significant threat for premature death. Current methods evaluate cardiotoxic damage based on volumetric changes in the Left Ventricle Ejected Fraction (LVEF). Indeed, a 10% drop in LVEF is commonly used as indicator of cardiotoxicity. More recently, a number of novel techniques have been developed that significantly improve specificity and sensitivity of heart’s volumetric changes and early detection of cardiotoxicity even in asymptomatic patients. Among these, the Strain by Speckle Tracking (SST) is a technique based on echocardiographic analysis that accurately evaluates myocardial deformation during the cardiac cycle (ventricular and atrial function). Studies also suggest that Magnetic Resonance Imaging (MRI) is a high-resolution technique that enables a better visualization of acute cardiac damage.

**Methodology:**

This protocol will evaluate changes in SST and MRI in cancer patients that received thoracic RT. Concomitantly, we will assess changes in serum biomarkers of cardiac damage in these patients, including: high-sensitivity cardiac Troponin-T (hscTnT), N-Terminal pro-Brain Natriuretic Peptide (NTproBNP) and Circulating Endothelial Cells (CECs), a marker of endothelial dysfunction and vascular damage.

**Discussion:**

The presented protocol is to our knowledge the first to prospectively and with a multimodal approach, study serological and image biomarkers off early cardiac damage due to radiotherapy.

With a practical clinical approach we will seek early changes that could potentially be in the future be linked to clinical mayor events with consequences for cancer survivors.

**Supplementary Information:**

The online version contains supplementary material available at 10.1186/s12885-021-08823-3.

## Background

In the last decades non-communicable diseases have increased, being responsible for about ¾ deaths worldwide. Among these, cardiovascular disease and cancer contribute with 2/3 of them - about 25 million deaths per year [1].

In the United States 15 and 14 million people are estimated with cardiovascular disease (CVD) and a history of cancer, respectively, and this number is rising as the population grows older and better therapies enhance longevity [2]. [3]. Although these diseases are usually thought as 2 independent entities that share similar risk factors, more recent research has proposed mechanisms of cross talk between cancer and cardiovascular disease [4].

Major advances in recent years have expanded the number of available treatments for patients with cancer. Despite this, radiation-therapy or radiotherapy (RT) remains as the most effective, non-surgical curative treatment against the disease; RT employs high-energy radiation to reduce tumor size by damaging cells’ DNA or generating free radicals that eventually damage and destroy cancer cells. In general, about 50% of all cancer patients receive some type of RT during their course of treatment, within this group approximately 60% receive RT with a “curative intent”, meaning the goal is the eradication of the tumor or preventing its future recurrence. Finally, RT is a highly cost-effective methodology, representing only a small fraction of the total cost of cancer care.

### Side effects of thoracic radiotherapy: cardiotoxicity & cardio-oncology

Despite its demonstrated effectiveness against cancer RT damages not only malignant cells but also normal cells within our bodies. Consequently, like any other cancer therapy it has side effects that can range from moderate to severe [5]. In particular, thoracic RT has well-documented side-effects upon the cardiovascular system [6], which are commonly known as “cardiotoxicity”. Early studies demonstrated that the exposure to ionizing radiation causes a dose-dependent cardiac damage that may result in a premature death of the patient due to ischemic events, cardiac valve failure/disorders or pericardial injury [7]. In recent years, the prevention, assessment and clinical management of cardiotoxicity in oncological patients has established the basis for a new discipline and an emerging field in oncological medicine called “cardio-oncology” [8]. As described above, radiation to the chest has the potential to cause damage to the heart in a dose-dependent manner. Subsequently, post-RT cardiac dysfunction can progress into heart failure via a complex mechanism that involves damage to the endothelium and microvascular injury [9, 10]. This protocol will assess the dose-dependent cardiotoxic effects of thoracic RT in cancer patients.

### Most cardiotoxic effects of thoracic RT manifest years, even decades after exposure

Unlike other cancer therapies that can also cause cardiotoxicity (including anthracyclines & chemotherapeutics), RT-derived cardiac damage is usually manifested after years or even decades from initial exposure [ [11]]. Indeed, studies demonstrate that exposure to radiation can damage the intimal layer of blood vessels also triggering atherogenesis, and over the years these can lead to ischemia. Concomitantly, the damage to small vessels can also affect cardiac contractility [12]. Unfortunately, these effects develop over a prolonged period of time and become clinically apparent only after the cardiac performance is compromised and the reduction in the patient’s heart function can be measured, difficulting its diagnosis. At present, the sensitivity and specificity of the tools employed to measure cardiac function are somewhat imperfect and sometimes over-estimate or under-estimate RT-derived cardiotoxicity.

### Strain by speckle tracking (SST) as an early marker of cardiac damage and dysfunction

**Fig. 1:**
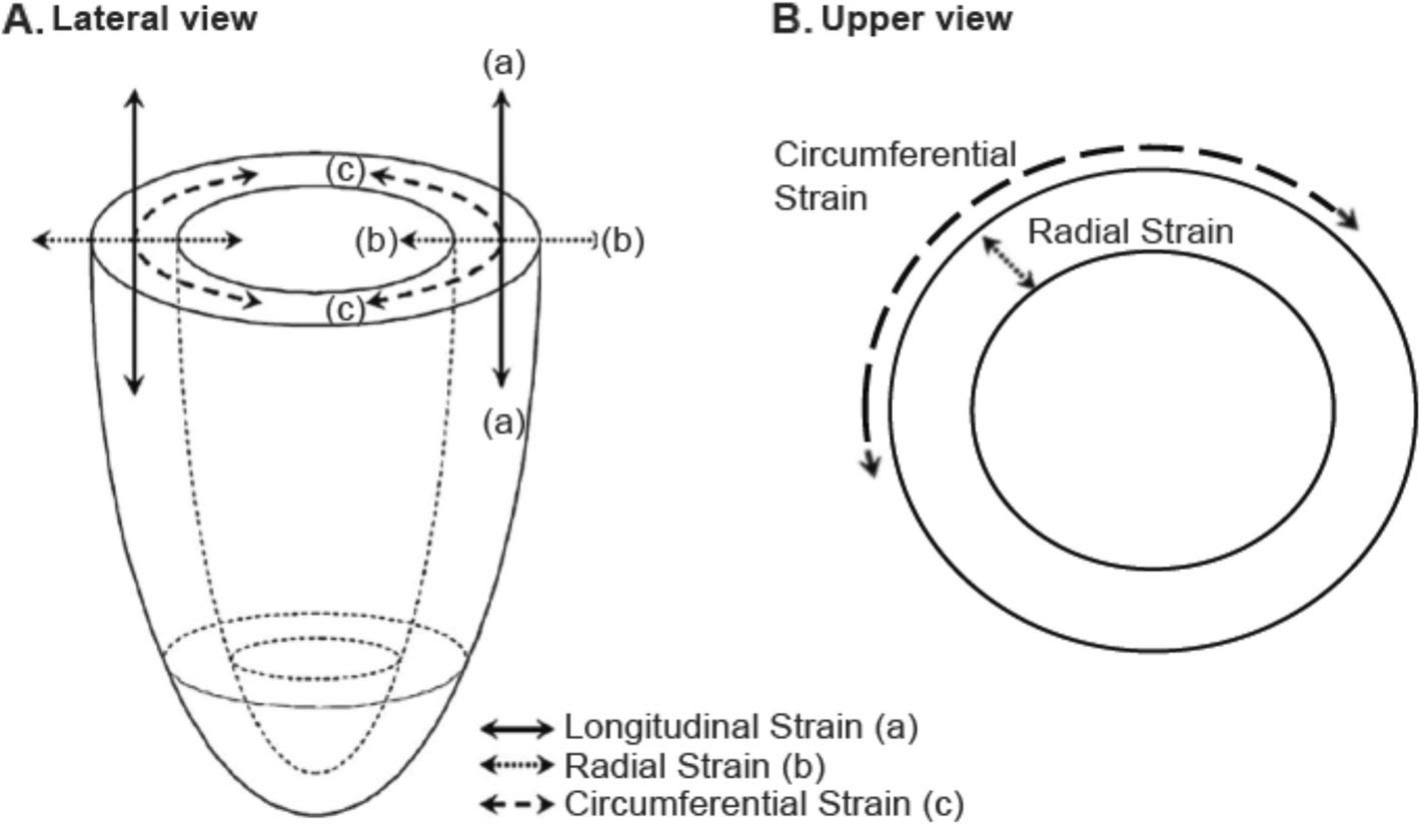
Schematic representation of different components of myocardial deformation measured by Strain Speckle Tracking (SST). Figure shows a simplified diagram of a lateral (**A**) and upper view (**B**) of a ventricle. Basic types of strain are displayed as (**a**), (**b**) and (**c**). See text for further details. Modified from Bansal and Kasliwal (2013)

For years the echocardiogram has been the standard tool to assess cardiotoxicity in cancer patients subjected to thoracic RT. By this method, cardiac function is estimated based on volumetric changes of the left ventricle during the cardiac cycle by Simpson’s method, commonly known as Left Ventricular Ejection Fraction (LVEF) a parameter that is affected by pre and post load conditions. Thus, a 10%-drop in LVEF has been traditionally considered as a parameter to define “cardiotoxicity”. However, as previously pointed out this is a rather “late” event in the progression to heart failure, and by the time these changes occur there are structural modifications on the myocardium often irreversible, or difficult to compensate at best. Recently, a variety of novel echocardiographic techniques have been developed; these are largely based on the transient deformation (shortening) of the myocardial fibers assessed with changes of the “speckle” pattern observed in an echocardiogram during a normal cardiac cycle. Although myocardial deformation during the cardiac cycle is a multidimensional process it can be simplified into three basic strains [13] as shown by Fig.1 (Modified from Bansal) for analysis: (a) longitudinal, (b) radial and (c) circumferential. -Longitudinal strain refers to the shortening of the myocardial fibers along its major axis (longitudinal). -Radial strain denotes the thickening of ventricle wall in reference to its radius -Circumferential strain relates to a reduction in the circumference of a heart cavity (ventricle or atrium) during the cardiac cycle. Figure 1 shows a simplified diagram of the components of myocardial deformation that can be assessed with Strain by Speckle-Tracking echocardiogram (hereafter, simply called SST). In summary, the use of the SST allows a more accurate and much earlier assessment of alterations in myocardial contractility compared to the traditionally used 10%-drop in LVEF and thus previous studies have assessed RT-induced acute myocardial damage by SST in multiple settings and has been suggested as an early indicator of subclinical cardiac dysfunction [14–16]. Furthermore, SST alterations predict mortality among acute heart failure patients [17]. Hence, one of the main goals of this proposal is to evaluate SST patterns as early predictors for the development of cardiotoxicity and acute damage during or after thoracic RT treatment across different dosages.

### Magnetic resonance imaging (MRI) is a high-resolution technique that allows assessment of acute cardiac damage by RT treatment

Studies demonstrate that MRI provides a higher spatial resolution to accurately visualize myocardial lesions compared to other techniques such as scintigraphy [18]. A study in esophageal cancer patients that received high doses of RT (>60Gy) used MRI to demonstrate that RT induces cardiac fibrosis and edema [19] suggesting this is an effective technique to assess RT-induced cardiac damage. Therefore, our proposal will incorporate MRI analysis pre- and post-RT in cancer patients as an imaging assessment along with SST. MRI will include Cardiac Magnetic Resonance Cinema Imaging (Long axis balance, Balance 4 cameras, Short shaft full balance, Right ventricular balance), Anatomical imaging (Inversion recovery single shot balance, 3D short axis covering the entire heart and aorta with free respiratory trigger), Flow Imagining (2D outflow tract of the aorta and 2D pulmonary artery outflow tract) and Quantitative Imaging (T1 map short axis apical section, T1 map short axis medial section, T1 map short axis basal section, T2 map short axis apical section, T2 map short axis medial section, T2 map short axis basal section).

### Other soluble/biochemical factors may also serve as early predictors of cardiotoxicity

In addition to alteration in the SST patterns or MRI, a number of soluble factors have been postulated as markers for cardiac dysfunction and will be assessed as early predictors of cardiotoxicity:

1. High-sensitivity cardiac troponin-T (hscTnT): Troponins are key regulators of cardiac contractility by controlling the calcium-mediated actin-myosin interaction. More specifically, soluble levels of cardiac troponin-T have been traditionally used as a marker for myocyte injury and cardiac dysfunction in acute and chronic heart failure. More recently, a high-sensitivity assay that efficiently detects low cardiac troponin-T concentrations has been developed [20]. Originally used to diagnose acute coronary syndrome, the high-sensitivity cardiac Troponin-T (hscTnT) has gained attention for its predictive value upon cardiovascular disease. In fact, a recent study demonstrated that increasing cardiac radiation doses, given as adjuvant RT, were associated to a rise in hscTnT [21]. Furthermore, elevated hscTnT levels can identify cancer patients with a higher risk to develop cardiac complications derived from anthracycline treatment [22], which, like thoracic RT, can be associated to cardiotoxicity.
2. N-Terminal pro-Brain Natriuretic Peptide (NTproBNP): Brain Natriuretic Peptide (BNP) and its pro-hormone the N-Terminal pro-BNP (NTproBNP) are mainly secreted by cardiomyocytes located in the heart’s left ventricle in response to stretching caused by increases in cardiac filling pressure or volume load. Ventricle or atrium secreted BNP and NTproBNP have been successfully used as markers to diagnose congestive heart failure. Similarly to hscTnT, NTproBNP levels have been used to identify patients at higher risk for cardiac dysfunction; a study demonstrated a correlation between elevated NTproBNP and a reduction of fractional shortening measured by echocardiogram in asymptomatic cancer patients that received anthracyclines. The study proposes NTproBNP as an early serum marker for anthracycline-induced cardiotoxicity [23].

### Circulating endothelial cells (CECs) as early predictors of RT-derived cardiotoxicity

Circulating Endothelial Cells (CECs) are a subpopulation of fully-differentiated mature endothelial cells that have detached from the vascular walls. This may occur under certain circumstances such as tumor angiogenesis, derived from smoking, by acute myocardial infarction [24] or hypertension [25]. Although CECs were described over 30 years ago, they were only recently recognized as a reliable marker for endothelial dysfunction [26], and studies have postulated their role on the pathogenesis of ischemic vascular disease [27]. In particular, apoptotic CECs are predictors of cardiac vasculopathy in heart transplanted patients [28] and are well established as indicators of vascular injury and endothelial damage. On the other hand, RT is known to produce endothelial vascular injury through a variety of mechanisms [29]. Hence, our proposal will assess changes in apoptotic CECs prior and after RT in cancer patients. We hypothesize that high-RT doses will increase apoptotic CECs. Our proposal will compare imaging by SST and MRI against soluble/biochemical markers as early predictors of cardiac dysfunction in RT-treated cancer patients.

### Measuring the impact of high & low doses of RT on cardiotoxicity

**Fig. 2:**
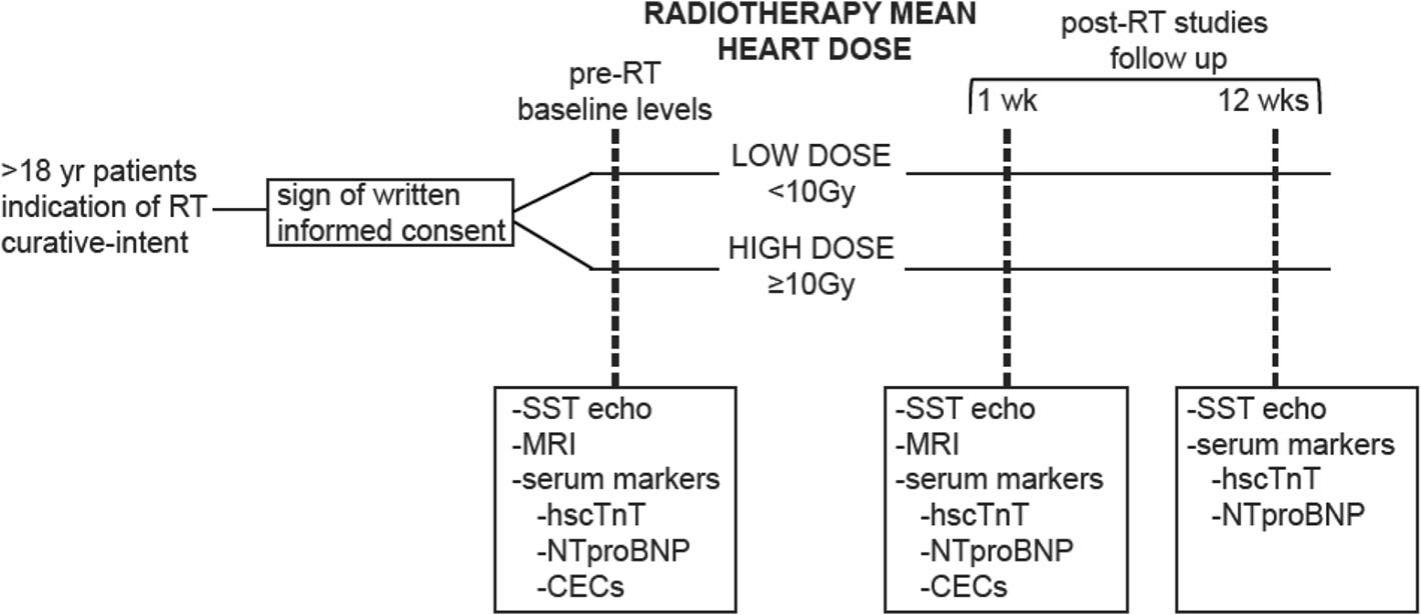
Three-year work plan

As explained above cardiotoxic damage by RT is dose dependent. Evidently, the success of a RT treatment in eradicating a tumor is determined by the total radiation dose given measured in Gray (Gy), however, this is limited by the tolerance of the surrounding normal tissue [5]. Similarly, previous studies have demonstrated that cardiotoxicity effects are proportional to the radiation dose applied to the patient. In order to quantify the dose-dependent effects of thoracic RT we will stratify our patients into two categories according to the calculated mean heart dose received. Therefore, our proposal will stratify patients receiving low (<10Gy) and high (>10Gy) doses of RT (see Fig. 2 and the methodology section for further details).

### Relevance of the proposal

In recent decades, novel more effective therapies against cancer have significantly prolonged patient survival. Consequently, the number of cancer survivors with “late” anti-cancer treatment derived complications is expected to rise in coming years. Among these complications, cardiotoxicity represents a significant risk for premature death [8]. It is estimated that approximately 50% of all oncological patients receive some type of RT during their treatment. As occurs with other therapies, thoracic RT is associated to a risk of developing cardiotoxicity in a percentage of patients. Unfortunately, current standard techniques only allow the detection of cardiac dysfunction at an advanced stage in the progression to heart failure (a 10% drop in LVEF). Here we propose the use of a combination of echocardiogram SST, MRI and serum factors associated cardiac/endothelial dysfunction as early predictors of late cardiotoxicity derived from RT treatment.

### Significance for patients

Estimates in the United States indicate that approximately 4 million cancer survivors die from cardiovascular disease derived complications. An earlier identification of patients at a higher risk for late thoracic RT-derived cardiotoxicity may help identify the best candidates for a risk adapted clinical follow-up in order to prevent long-term cardiac complications aiming to minimize the risk of death. Likewise, this will pave the way to testing new interventions to reduce cardiac damage, as it has been the case for anthracycline-induce cardiotoxicity. The SST echocardiogram is a relatively simple, low-cost, non-invasive assay that can be routinely performed by cardiologists in any clinical setting.

## Methods/design

- OBJECTIVES & HYPOTHESIS
- HYPOTHESIS
- Thoracic radiotherapy causes dose-dependent alterations in the Strain by Speckle Tracking (SST) that can be used as an earlier predictor of cardiotoxicity compared to serum biomarkers of cardiac dysfunction

### General goal

To evaluate and compare the changes in different modalities: Strain by Speckle Tracking (SST), MRI, soluble markers of cardiac dysfunction and apoptotic CECs as early predictors of cardiotoxicity in cancer patients receiving low or high mean heart doses of RT.

### Specific goals

- To prospectively enroll cancer patients scheduled to receive high and low doses of thoracic RT with curative intent.
- To assess imaging and cardiac parameters using SST and MRI in cancer patients prior and after thoracic RT.
- To evaluate plasma levels of hscTnT, NTproBNP and apoptotic CECs in cancer patients prior and after thoracic RT.

### Methodology

GOAL 1-To prospectively enroll cancer patients scheduled to receive high or low doses of thoracic RT with curative intent.

Patients: Inclusion criteria:

- ≥18 year-old patients with histologically confirmed thoracic cancer (breast, esophagus or lung) and recommendation of thoracic RT with curative intent with or without chemotherapy will be prospectively enrolled at the Cancer Center Red de Salud UC CHRISTUS. All patients must be able to read and understand Spanish in order to sign a written informed consent form.

Patients: Exclusion criteria:

- Patients with no medical records, pregnant, or those previously diagnosed with severe left ventricular dysfunction or a cardiac insufficiency prior to RT, patients with metastatic disease and indication of palliative RT or patients with significant comorbidities associated to a a < 5 year life expectancy. Finally, patients that had been previously treated with thoracic RT are also excluded.

### RT dosage: mean heart dose

The cardiovascular risk score ACC/AHA 2013 for every patient will be calculated prior to RT (previously validated by Acevedo et al. [30] RT planning for every patient will be elaborated following international guidelines. Heart volume will be contoured according to the atlas provided by Duane et al. [31]. Whenever, possible the most advanced equipment will be used to deliver the planned dose to the targeted tumor, minimizing the dose to the heart or other organs that may be at risk. Strategies employed may include: Intensity modulated RT (IMRT), volumetric modulated arc therapy (VMAT), or image guided RT (IGRT). For dosimetry planning and restrictions for the heart volume we will follow the criteria provided by Marks et al. [32].

Accordingly, patients will be stratified based on the mean heart dose received into two groups: Low mean heart dose (<10Gy) and high mean heart dose (≥10 Gy) (see Fig. 2). Patients will be treated in an Electa Axesse or Varian Clinac 2100, and planning will be done using the Monaco 5.11 or Eclipse with Montecarlo and Pencil beam algorithm. For adjuvant breast cancers standard Tangent technique will be used with Field-in-field to minimize hot spots in the target. Usual cardiac blocks will be used to reduce heart dose. 40–60 Gy in 15–30 fractions will be delivered. An additional Oblique supraclavicular field will be added for regional lymph node RT if needed. 6–18 Mv X-rays will be used. For neoadjuvant esophagus cancer VMAT technique will be used. 41.4 Gy in 23 fractions will be delivered to the target. 6 Mv X-rays will be used. A Cone beam CT (CBCT) prior to each treatment will used to verify setup. For definitive treatment, in esophagus or lung cancer 60 Gy in 30 fractions will be delivered with same technique and CBCT protocol. The medical oncologist will prescribe concomitant chemotherapy if required.

### Sample size

We anticipate a total n of 60 patients for this study. Patients will be categorized according to the diagram shown by Fig. 2 and distributed equally into groups: Low dose RT (*n* = 30), and high dose RT (*n* = 30) Sample size was calculated assuming a 30% change by SST in the group of patients receiving high-dose RT, an alpha = 5% and a statistical power = 80% (beta = 0.2). This gives a sample size of 28 patients. Baseline status (pre-RT) will be compared to 1 week following RT (post-RT). An additional 10% of patients will be recruited (*n* = 30) in order to avoid data loss during follow up.

Expected results We expect to enroll the proposed number of patients (*n* = 60 in total) during the first 12–24 months of the study in order to accomplish this goal (also see work plan).

GOAL 2-To assess imaging and cardiac parameters using SST and MRI in cancer patients prior and after thoracic RT.

### SST echocardiogram

The SST echocardiogram for consented patients is performed following the standard procedure in a medical attention box at the Cancer Center. The procedure lasts approximately 20 min, and is performed by a trained cardiologist using a portable device. Parameters measured include heart contractility, ventricular/atrial ejected fractions. Obtained data are used to calculate longitudinal, radial and circumferential strains as described above (see Fig. 1). Combined data are also used to calculate global longitudinal strain (GLS) using the average peak strain from 3 apical projections of the left ventricle. SST measurements will be recorded for every patient prior to RT, and also 1 and 12 weeks after completion of RT treatment as described in Fig. 2. Importantly, concomitant use of chemotherapy in patients will also be recorded. For analyses, the % of change in every patient will be assessed comparing levels before and after RT performing a paired analysis.

### Magnetic resonance imaging/cardiac magnetic resonance

High-resolution, non-contrast-enhanced cardiac nuclear magnetic resonance (CMR) scans will be performed at the Radiology and Diagnostic Imaging Unit of Red Salud UC-Christus (Santiago, Chile) on a Philips 3.0 T Ingenia Gyroscan MRI scanner equipped with Release 5.3 Clinical Packages (Philips Healthcare, Best, The Netherlands). Following safety questionnaire and signing of informed consent patients will be in supine position for research studies. Trained radiologists from the Radiology Department at the School of Medicine in the Pontificia Universidad Catolica de Chile will blindly review and inform the obtained MRI results. CMR scans will be performed at baseline (pre-RT), and after the administration of last fraction (post-RT) according to radiation therapy treatment plan.

The same imaging protocol will be used for pre and post RT measurements. The body coil will be used for signal transmission, and a 16-element anterior and 12- element posterior phased-array coil will be used for signal reception. Image acquisition will be performed as described [33], with: i) electrocardiography-gated, motion corrected, non-contrast-enhanced T1 mapping using the modified look-locker sequence (MOLLI) parameterized as follows: slice thickness 8.0 mm, field of view 300 × 400 mm, flip angle 50 degrees, minimum TI 120 ms, inversion-time increment 80 ms and sensitivity encoding (SENSE) factor 3. Freebreathing or breath-hold will be used according to patient’s characteristics. Additionally, ii) electrocardiography-gated, breath-hold-gated, steady-state free precession long-axis cine images in 2, 3, and 4-chamber views will be acquired parameterized as follows: slice thickness 8.0 mm, no gap, field of view 280 × 300 mm, 208 × 256 matrix, repetition time 2.9 ms, echo time 1.2 ms, flip angle 70 degrees, temporal resolution 0.05 indicating a normal distribution).

### Comparisons of low-dose (LD) versus high-dose (HD)-RT

1. Associations tests among clinical variables between these two groups will be performed (chi-square or Fisher’s exact). Depending on normality tests for age Student’s t-test or Wilcoxon test will assess age differences comparing LD versus HD
2. First, baseline assessment of SST (pre-RT). Baseline MRI and serum markers will also be assessed. Statistical significance will be set at 0.05 and will be included into a multivariate analysis. These will be contrasted to 1-week and 12-week measurements (post-RT)
3. Relative risk (RR) will be reported using a confidence interval at 95% for SST+ comparing LD versus HD. Also sensitivity analyses will be performed in order to assess potential confounders and RR will be reported for every subgroup

### Comparisons pre versus post-RT

1. Associations tests (chi-square or Fisher’s exact) will be performed in each group (LD and HD) among clinical variables and at various times (pre-RT, 1-week or 12-week post-RT), *p*-values < 0.05 will be considered statistically significant. Primary hypothesis will be confirmed when % of change in the HD group is ≥30% statistical significance will be set at *p* < 0.05.
2. First, we will compare SST at pre-RT versus 1-week post-RT, and pre-RT versus 12-week post-RT. Also, baseline MRI and serum markers will be compared to 1-week and 12-week post-RT. Significant differences (*p* < 0.05) will be included into the multivariate analysis.

### Multivariate analysis

A logistic multivariate regression analysis will determine SST at 1-week post-RT, other independent variables will be included: age, disease details, soluble markers status comparing LD versus HD. Analyses will also assess interaction or confusion among outcome associated variables on bivariate analyses. OR for these tests will be reported using a confidence interval of 95% Diagnostic yield of MRI versus standard SST. A diagnostic yield analysis comparing MRI against SST will report area under the curve (AUC), sensitivity, specificity, positive and negative predictors, and likelihood ratios (all at 95% confidence intervals). Finally, all analyses and calculations will use STATA v.12 (College Station, TX, StataCorp LP).

### Expected results

We expect increases in serum biomarkers of cardiac dysfunction after RT, especially in those patients receiving higher doses of radiation. Regarding statistical analyses we expect significant changes by comparing pre-RT versus post-RT levels in each case.

### Work plan

**Fig. 3:**
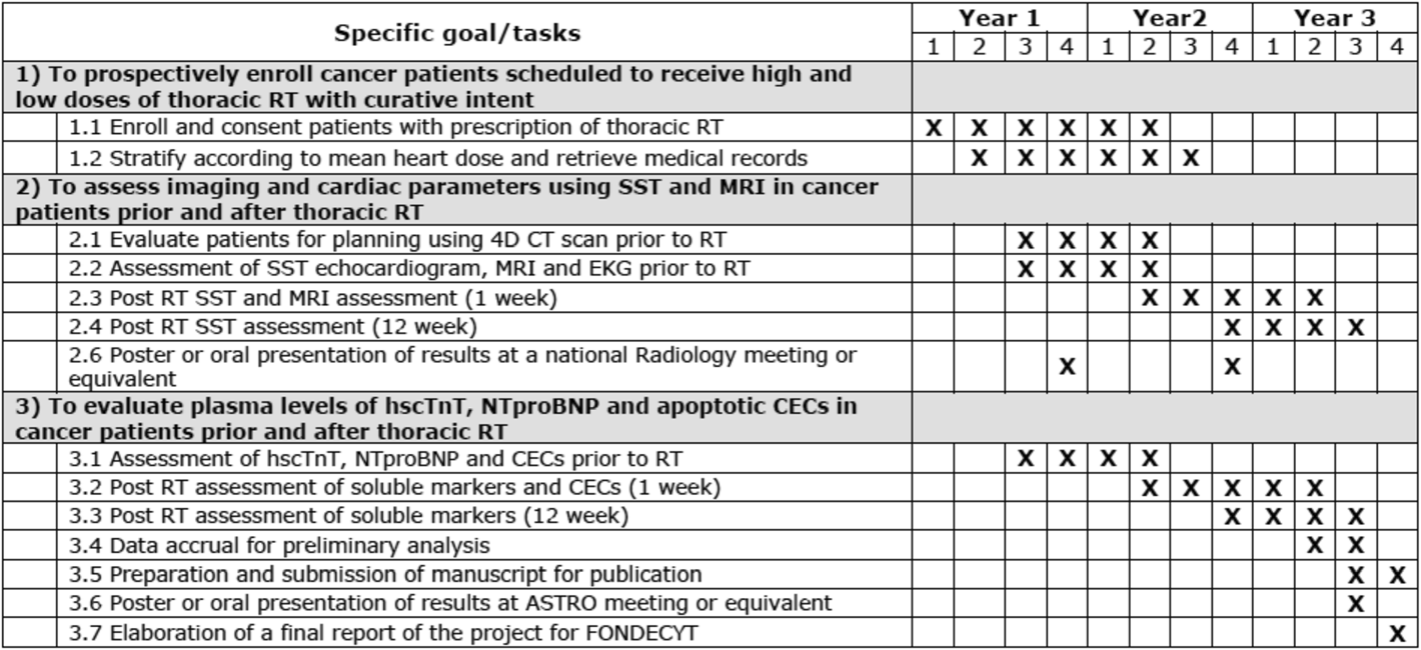
Patient timeline for this study. Briefly patients are selected according to inclusion/exclusion criteria and then stratified into two categories based on mean heart dose: low or high. Baseline levels of serum biomarkers are determined prior to RT and 1, and 12 weeks after finishing RT. Similarly Strain by Speckle Tracking (SST) echocardiogram are measured prior to RT and 1 and 12 weeks post RT. Please see text and methodology section for further details

Our 3-year work plan for this proposal is shown below and describes the main tasks for every specific goal of the project and the estimated time frame for publication of manuscripts and presentation at scientific meetings. The goals or specific tasks indicated are organized quarterly by every year of the proposal. (see Fig. 3). This protocol will follow the SPIRIT guidelines (See SPIRIT checklist in supplementary material).

## Discussion

The presented protocol is to our knowledge the first to prospectively and with a multimodal approach, study serological and image biomarkers off early cardiac damage due to radiotherapy.

With a practical clinical approach we will seek to detect early changes that could potentially be in the future be linked to clinical mayor events with consequences for cancer survivors.

With an early detection of subclinical damage, we could open a therapeutic window where cardioprotective interventions could be applied, modifying the natural history of late consequences of radiation to cardiac structures. Likewise, we expect that the present project will aid in the understanding of the physiopathology of this process and the identification of dosimetry and clinical predictors of mayor cardiovascular damage.

## Supplementary Information

**Additional file 1.** . SPIRIT 2013 Checklist: Recommended items to address in a clinical trial protocol and related documents*.

## Data Availability

The datasets used and/or analyzed during the current study are available from the corresponding author on reasonable request.

## Abbreviations

CVD: Cardiovascular disease
RT: Radiotherapy
SST: Strain by speckle tracking
LVEF: Left Ventricle Ejection Fraction
MRI: Magnetic Resonance Imaging
hscTnT: High-sensitivity cardiac troponin-T
NTproBNP: N-Terminal pro-Brain Natriuretic Peptide
CECs: Circulating Endothelial Cells
IMRT: Intensity modulated RT
CBCT: Cone beam CT
GLS: Global longitudinal strain
CMR: Cardiac Magnetic Resonance
VMAT: Volumetric modulated arc therapy
IGRT: Image guided RT
LD: Low-dose
HD: High-dose
RR: Relative risk
AUC: Area under the curve
